# Validation of the traditional Chinese version of the diabetes eating problem survey-revised and study of the prevalence of disordered eating patterns in Chinese patients with type 1 DM

**DOI:** 10.1186/s12888-023-04744-6

**Published:** 2023-05-31

**Authors:** Chi Wing Lok, Mei Cheung Wong, Kim Wai Yip, Wing Ka Ching, Edward Kwok Yiu Choi

**Affiliations:** 1 Present Address: Department of Psychiatry, United Christian Hospital, Kowloon, Hong Kong; 2 Lingnan University, New Territories, Hong Kong

**Keywords:** Disordered eating behaviours, Disordered eating patterns, Type 1 diabetes mellitus, T1DM, Diabetes eating Problem Survey-Revised, DEPS-R

## Abstract

**Background:**

Disordered eating behaviours (DEBs) in patients with type 1 diabetes mellitus (T1DM) are associated with an increased risk of complications and mortality. The Diabetes Eating Problem Survey-Revised (DEPS-R) was developed to screen for DEBs in T1DM patients. The objectives of this study were to develop a traditional Chinese version DEPS-R (electronic version) and to measure the prevalence of DEBs in a local population sample.

**Methods:**

The DEPS-R was translated into traditional Chinese, modified and developed into an electronic version. The psychometric properties of the C-DEPS-R were tested on T1DM patients from 15 to 64 years old. The factor structure of the traditional C-DEPS-R was examined by confirmatory factor analysis (CFA). The C-EDE-Q and the C-DES-20 were used for convergent and divergent validity testing, respectively. Module H of the CB-SCID-I/P was used as a diagnostic tool for eating disorders. A correlation study was conducted with the C-DEPS-R scores obtained and the clinical characteristics. Type 2 diabetic (T2DM) patients on insulin treatment were recruited as controls.

**Results:**

In total, 228 T1DM patients and 58 T2DM patients were recruited. There was good internal consistency of the traditional C-DEPS-R (electronic version), with the McDonald’s omega of 0.825 and test-retest reliability of 0.991. A three-factor model of the traditional C-DEPS-R was confirmed by CFA. The cut-off score for the traditional C-DEPS-R was determined to be 24; 13.2% (95% CI 8.8%-17.5%) of T1DM patients were found to score above the cut-off score, while 7.5% (95% CI 4-10.9%) scored above the cut-off by the C-EDE-Q, and 4.4% (95% CI 2.1%-7.9%) were diagnosed with eating disorders by the CB-SCID-I/P Module H. Females with T1DM scored higher on the traditional C-DEPS-R. There was a significant correlation of the C-DEPS-R with BMI, occurrence of DKA, use of a continuous glucose monitoring system and positive diagnosis by the CB-SCID-I/P module H (p < 0.05).

**Conclusion:**

The traditional Chinese-DEPS-R (electronic version) demonstrated good psychometric properties. It is a self-rated, time-efficient and reliable tool for the screening of disordered eating behaviours in T1DM patients in the Chinese population of Hong Kong.

**Plain English Summary:**

Disordered eating behaviours, such as insulin omission, are associated with an increased risk of diabetes mellitus-related complications and mortality. Generic screening tools for eating disorders may over- or underestimate such problems in diabetic patients. Type 1 diabetes mellitus patients are at particular risk of developing disordered eating behaviours or eating disorders, yet studies in Chinese populations are limited. This study developed and validated the traditional Chinese (electronic) version of the Diabetes Eating Problem Survey-Revised (DEPS-R). The traditional Chinese-DEPS-R is a self-rated, time-efficient and reliable tool for the screening of disordered eating behaviours in Type 1 diabetes mellitus patients in the Chinese population of Hong Kong. The study also estimated the prevalence of disordered eating behaviours in diabetic patients from the local Chinese population, and the clinical correlations of the symptoms and clinical parameters were explored. The study reflected a higher prevalence of eating problems in the Type 1 diabetes mellitus population and demonstrated significant correlations of eating problems with BMI as well as the occurrence of diabetic ketoacidosis. Correspondence: lcw891@ha.org.hk.

**Supplementary Information:**

The online version contains supplementary material available at 10.1186/s12888-023-04744-6.

## Introduction

In all populations, eating disorders (EDs) pose a serious threat to a person’s health and well-being. EDs are associated with medical complications [1, 2] and psychiatric comorbidities [3]. From 1990 to 2019, the prevalence rate of anorexia nervosa (AN) increased by 1.6% (95% CI 1.5–1.6%), and the prevalence rate of bulimia nervosa (BN) increased by 2.0% (95% CI,2.0-2.1%) in China [76]. In Hong Kong, 3.9% of males and 6.5% of females aged 10–21 years in a sample of 2,382 students suffered from disordered eating assessed by the Eating Attitudes Test-26 [77]. EDs or disordered eating behaviours (DEBs), particularly among the diabetes mellitus (DM) population, are of concern to health care providers.

Diabetes mellitus (DM) is a chronic disease where not enough insulin is produced by the pancreas or when one’s body cannot effectively make use of produced insulin. The majority of diabetic patients suffer from type 2 diabetes (T2DM) where the body cannot effectively make use of the insulin it produces. There is proposed inter-relationship between T2DM and ED. T2DM patients on diet control or being overweight have increased risk of developing ED, while on the other hand, ED induced body weight gain which increases risk of one’s development of T2DM [78]. Another type of diabetes is type 1 diabetes (T1DM), where one’s autoimmune system attacks its own β-cells in the pancreas, which secretes insulin; thus, patients need to depend on insulin injections for blood glucose control [37].

Eating problems pose particular risks to type I DM (T1DM) patients who depend on insulin. As a form of disordered eating behaviour (DEB), T1DM patients deliberately omit or manipulate insulin doses as a unique way of purging [4]. In addition to insulin omission, other DEBs or disordered eating patterns, including purgative practices, food binging or restriction behaviours that are less frequent or severe enough to meet the diagnostic criteria for ED, have been identified in T1DM patients [5].

Patients with T1DM are more vulnerable to developing DEBs [6, 7]. The Modified Dual Pathway model suggests reasons for the risk of EDs in T1DM patients, including DM diet control, weight gain due to the initiation of insulin and the extra calories consumed to avoid hypoglycaemic attacks [8]. Several studies in DM patients revealed higher chances of DEB in T1DM patients. The reported prevalence of eating problems in T1DM varies from approximately 14-37% for DEB [9, 10] and approximately 10–33% for clinical ED [9, 11, 12]. In a meta-analysis [13], more DEBs and EDs were found in adolescents with T1DM than in nondiabetic adolescents.

T1DM patients with DEBs have a greater likelihood of developing complications from [12, 14, 15]. An 11-year follow-up study on 234 women with T1DM found a threefold increase in mortality rate for patients who had restrictive use of insulin [16].

### Psychopathology and proposed disease models

From the transdiagnostic perspective, diagnoses of AN, BN and BED carry similar underlying core psychopathological features. There is disturbance over the way one experiences one’s body shape, with identity difficulties. Patients with EDs suffer from a dysfunctional system of evaluating self-worth through their eating habits, body shape and weight, usually with predisposed identity issues of perfectionism and low self-esteem. The psychopathology of generic EDs and that of DEBs in T1DM were similar with respect to identity issues and body shape and weight concerns, also with a coincident peak onset age.

The psychopathology of DEBs in T1DM differs from that of the generic EDs over the DM-related effects on the illness model. The psychopathology of DEB in T1DM patients was proposed by different disease models; for example, the modified dual pathway model [8] suggests that T1DM is associated with DEB via three mechanisms: (1) carbohydrate counting-imposed food preoccupation, (2) weight fluctuations associated with variable use of insulin and subsequent body dissatisfaction, and (3) blood glucose fluctuations associated with mismatched insulin doses, excessive caloric intake secondary to hypoglycaemia, and the resultant weight gain. T1DM patients are also predisposed to developing ED because of inadequate coping styles or disordered family functioning. The initiation of insulin therapy can cause weight gain and subsequent emotional distress, which can lead to DEBs and continue a vicious cycle [17]. De Paoli & Rogers [18] proposed the transdiagnostic model of disordered eating in T1DM. The transdiagnostic model of disordered eating in T1DM suggests that low self-esteem and perfectionism predispose T1DM individuals to a dysfunctional self-evaluation of their eating habits, weight and body shape [18]. T1DM individuals may be further pressured by the learned importance of diet restriction and glycaemic control. The uncertainties and frustration of diabetes control may pose risks of developing DEB, while dietary restriction may also be associated with binging. In addition, there is a higher propensity for disinhibited eating due to hypoglycaemia [19].

As DEB poses a risk of physical complications and mortality in T1DM patients [20], early detection of DEBs is crucial. Currently available ED assessment tools include structured interviews, e.g. Eating Disorder Examination (EDE) [21]; and self-reported assessments, e.g. The Eating Disorder Inventory (EDI) [22] and The Eating Disorder Examination Questionnaire (EDE-Q) [23] are some of the commonly adopted tools for eating disorders. However, these assessments tools do not take insulin restriction into account and may misinterpret diet restriction and carbohydrate counting in DM care. This can cause both over- or underestimation of DEBs and EDs in people with T1DM [24–26]. For early detection of DEB in T1DM, a specific tool was designed for use in screening DEB in T1DM [27].

### The diabetes eating Problem Survey–Revised (DEPS-R)

The Diabetes Eating Problem Survey–Revised (DEPS-R) is a self-reported screening tool for assessing DEB in patients with T1DM. The instrument originally consisted of 28 items and was revised to 16 items. It scores on a Likert scale from zero (never) to five (always), with total scores ranging from 0 to 80 [27]. The cut-off score was defined at 20. A higher score indicates more DEB and greater pathology in the individual being tested. The DEPS-R can be completed in less than 10 min and has demonstrated good psychometric properties. The DEPS-R was validated in adolescents with T1DM [27], and demonstrated internal consistency with Cronbach’s alpha of 0.86; it was also validated in adults (18–79 years old) with T1DM [28] and demonstrated a Cronbach’s alpha of 0.84.

The DEPS-R was translated and validated in different versions, including German [29] French [30], Italian [31], Norwegian [28], Turkish [32] and Greek [33]. It has been recently translated and validated into Mandarin Chinese (simplified Chinese) [34], in which the study explored the factor structure of the DEPS-R, yielding three factors tapping into maladaptive eating habits, preoccupation with thinness, and maintaining high blood glucose values to lose weight. Factor 1 contained nine items tapping into maladaptive eating habits, e.g. “I skip meals and/or snacks” “When I overeat, I don’t’ take enough insulin to cover the food” “After I overeat, I skip my next insulin dose.”. Factor 2 contained four items tapping into preoccupation with thinness, e.g. “Losing weight is an important goal to me.” “ I would rather be thin than have good control of my diabetes.” and Factor 3 contained three items looking for maintenance of high blood glucose values to lose weight, e.g. “I try to keep my blood sugar high so that I will lose weight.” All item loadings were greater than 0.4 among youths and adults with T1DM.

There are a few reasons behind for developing the traditional Chinese version of the scale. The majority (88.8%) of citizens in Hong Kong are Cantonese users (traditional Chinese in written language) [35]. In view of the significant differences in the linguistic and cultural context of Mainland China and Hong Kong [36], a traditional Chinese version of the DEPS-R is required for local use. In addition, the Hong Kong citizens’ diet patterns are different from that of the Mainland Chinese. Hong Kong diet mostly composed with fast food, and processed or refined food due to hectic lifestyle and the demand for “efficient meals”, these foods are usually high in fat, sugar and other additives. The DM education and supports are also more established and systemic in Hong Kong, especially in the public health sector of the hospital authority.

## Objective

The objective of this study was to develop and validate the traditional Chinese version of the DEPS-R (C-DEPS-R) by examining the reliability and validity of the scale in Hong Kong Chinese adults and adolescents suffering from T1DM who were attending specialised DM outpatient clinics of the two hospitals in the Kowloon East cluster (KEC) in Hong Kong. It is hypothesised that the traditional C-DEPS-R has a three-factor structure and the questionnaire is valid and reliable to use in the Hong Kong Chinese T1DM population.

The other objectives were to explore the prevalence of DEBs among Hong Kong Chinese T1DM patients and the possible clinical correlations of DEBs measured by the C-DEPS-R with the clinical characteristics and DM control.

## Methodology and research design

### Development of the traditional chinese version of the DEPS-R (C-DEPS-R)

**Fig. 1:**
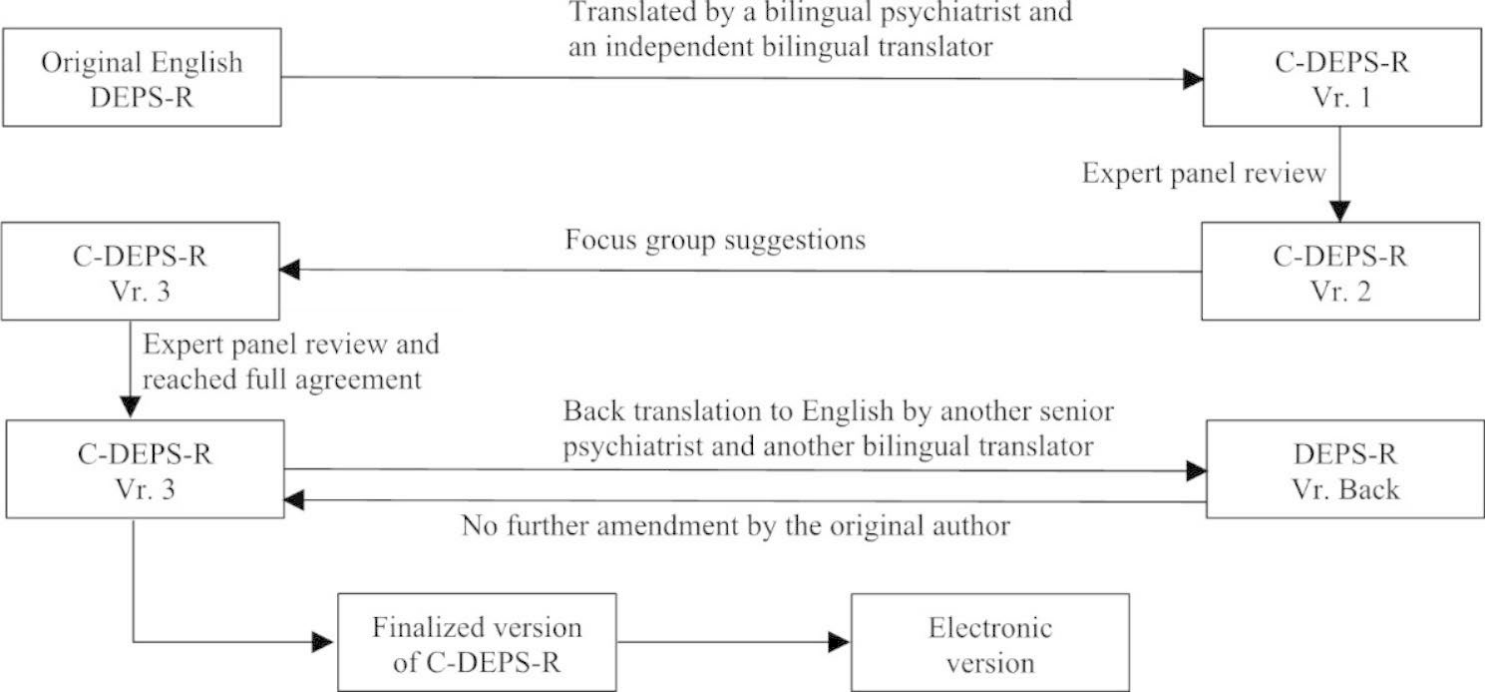
Development of traditional C-DEPS-R Note: DEPS-R: Diabetes Eating Problem Survey-Revised. C-DEPS-R: Traditional Chinese Diabetes Eating Problem Survey-Revised. C-DEPS-R Vr. 1: C-DEPS-R version 1. C-DEPS-R Vr. 2: C-DEPS-R version 2. C-DEPS-R Vr. 3: C-DEPS-R version 3.

Permission for translation into Chinese as well as validation was granted by the original author of the DEPS-R, Professor Lori Laffel, Professor of Paediatrics, Harvard Medical School and on behalf of the Joslin Diabetes Center. Approval for the study was granted by the Clinical and Research Ethics Committee of the Kowloon Central/Kowloon East cluster of the Hospital Authority of Hong Kong. Figure 1 shows the development of the traditional C-DEPS-R.

DEPS-R Vr. Back: DEPS-R back-translated version.

The content validity and comprehensibility of each item of the C-DEPS-R were reviewed by an expert panel comprising two senior psychiatrists: a consultant endocrinologist, a nurse consultant, an occupational therapist in the mental health service, a clinical psychologist and a dietitian. The items of the scale were modified based on the suggestions and discussion among the members. A pilot sample of 20 out-patients with T1DM were recruited from the out-patient DM clinic of both medical and paediatrics departments to test the applicability of the C-DEPS-R. The pilot run aimed to test the practicability of the logistics and the use of electronic scales.

### Recruitment of participants

Participants were recruited from the DM clinics of the Department of Paediatrics & Adolescent Medicine and the Department of Medicine of both the United Christian Hospital (UCH) and the Tseung Kwan O Hospital (TKOH) DM clinics in the KEC. All suitable T1DM patients were recruited for this study. T2DM patients on insulin were also recruited as a comparison group to explore whether patients of the two most common types of DM differ in prevalence of DEB. T2DM differs from T1DM by being non-insulin-dependent and with older age of onset [37], the pathogenesis of concurrent DEB and T2DM differ from that of concurrent DEB and T1DM by the sequence of the illness. The recruitment period was from 15 to 2020 to 15 May 2021. All patients followed up within the period were retrieved from the clinical system and were carefully reviewed based on the inclusion criteria: (1) Chinese ethnicity; (2) age of 15 to 64 years; (3) educational level at junior secondary school or above; (4) T1DM and T2DM patients on insulin as controls; (5) diagnosis of DM for at least 6 months; and (6) available informed written consent. Patients were excluded if they (1) were not able to read Chinese or input the electronic survey; (2) had other medical illnesses affecting diet intake or body weight, including but not limited to active thyroid diseases, neurological diseases, movement disorder, or major gastrointestinal illness, e.g., inflammatory bowel disease or post bariatric surgery status; (3) had an intellectual or cognitive disability; or (4) had undifferentiated or other types of DM (e.g., gestational DM).

The sample size was estimated according to the requirement of confirmatory factor analysis (CFA) [38]. After screening for inclusion and exclusion criteria, all suitable T1DM patients from the DM clinics of UCH and TKOH were recruited. Age-matched T2DM patients on insulin treatments from the DM clinics were recruited as controls via stratified randomisation with computer algorithms.

A total of 527 patients on insulin were identified by the computer systems of the DM clinics of TKOH and UCH within the study period from 15 to 2020 to 15 May 2021. T1DM and T2DM patients in the lists were identified and confirmed to be using insulin. Eight patients with other types of DM (including gestational DM, post-pancreatectomy or pancreatitis DM and undifferentiated types) were excluded. A total of 296 T1DM patients were identified. Twenty-eight T1DM patients were excluded according to the exclusion criteria. Fourteen T1DM patients failed to attend the follow-up appointment. Eight patients were excluded upon interview due to having attained an educational level below secondary school, while 18 patients refused to participate in the study.

**Fig. 2:**
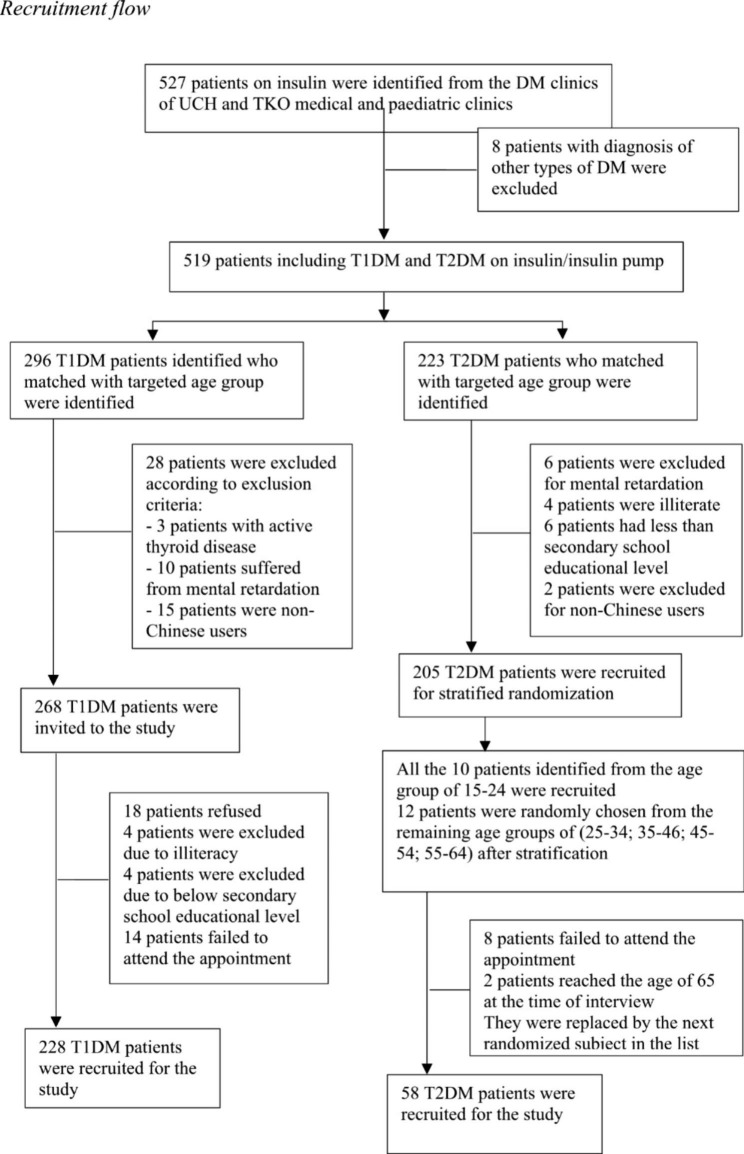
Recruitment flow

A total of 223 T2DM patients on insulin within the targeted age group were identified. Eighteen patients were excluded according to the exclusion criteria. Randomisation was performed with a computer program in each stratified age (aged 15–24; 25–34; 35–44; 45–54; 55–64 years), and 12 patients were randomly chosen from each of the stratified age groups except for the 15- to 24-year-old group, in which all 10 patients were invited without randomisation. The recruitment response rate was 94.1%, and the refusal rate was 5.92%. No significant difference was found when comparing the age and sex of the participants and the nonparticipants (Fig. 2). T1DM participants were invited to join the re-test, and 34 T1DM participants agreed to participate in the re-test after three to four weeks’ time.

### Measurements

*The BMI (Body Mass Index) was calculated from the clinical measurement of body weight and height on the day of recruitment assessment. The traditional C-DEPS-R was conducted as an electronic version and was compared with the Chinese-Eating Disorder Examination Questionnaire (C-EDE-Q) (electronic version), which is one of the standard tools for the assessment of eating disorders* [39], to assess the convergent validity of the scale. Module H (eating disorder) of the Structured Clinical Interview for DSM-IV Axis I disorders (CB-SCID-I/P) was used as a diagnostic interview for recruited patients. The C-DEPS-R was compared with the Chinese Diabetes Empowerment Scale (C-DES-20, which measures DM patients’ self-efficacy and ability to engage in challenges faced during DM control [40], to assess the divergent validity. Participants were invited to complete questionnaires with both written (C-DES-20) and electronic (C-DEPS-R and C-EDE-Q) versions. The participants were also interviewed with the use of a signed consent form.

The following measures were administered along with the interview and electronic version of the C-DEPS-R:

#### The Chinese Bilingual version of the structured clinical interview for dsm-iv-tr axis i Disorder, Research Version, Patient Edition (CB-SCID-I/P)

The Chinese-Bilingual Structured Clinical Interview for DSM-IV-TR Axis I, Patient version (CB-SCID-I/P) is a translated Chinese version of the SCID-I/P [41, 42].

#### The chinese version eating disorder examination questionnaire (C-EDE-Q)

The Eating Disorder Examination Questionnaire (EDE-Q) is a 28-item self-report survey for screening eating disorder psychopathology, which is based on the Eating Disorder Examination Diagnostic Interview and focuses on symptoms that have occurred within the previous 28 days. A mean score of four or above on any subscale or global score indicates a greater likelihood of clinical symptoms. All subscales demonstrated good internal reliability with a Cronbach’s alpha above 0.77 [43]. It was previously translated and validated in Hong Kong (electronic version) [44].

#### The chinese diabetes empowerment scale (C-DES-20)

The Chinese Diabetes Empowerment Scale (C-DES-20) is a 20-item self-report survey. It measures one’s perceived self-efficacy in DM care, which is related to the willingness and ability of an individual with DM to engage in challenges during disease management [40]. It was validated in Hong Kong in T1 and T2DM patients [45].

#### Psychometric properties of the C-DEPS-R

##### Reliability

The reliability of a test refers to the degree to which the same consistent measurements can be repeated over time. The reliability of the C-DEPS-R was established by calculating the internal consistency and the test-retest reliability. The internal consistency, which is reflected by McDonald’s Omega, indicates the extent to which items in the scale are homogeneous. The McDonald’s omega is applied due to skewed data distribution. A McDonald’s Omega values > 0.7 is expected to demonstrate internal reliability [66]. Test-retest reliability refers to the repeatability of the test scores over time. The C-DEPS-R was filled in by the same patient recruited two times after three to four weeks, so as to avoid major changes in the clinical conditions or retention of the previous answers. The test-retest reliability was assessed with the Wilcoxon signed-ranked test and Spearman’s Rho correlation coefficient. A Wilcoxon signed-rank test with p > 0.05 and Spearman’s rho correlation coefficient of over 0.7 are considered satisfactory [79].

##### Validation

Validity refers to how accurately a test measures what it is intended to measure. Content validity is the degree to which the content of an instrument adequately reflects the construct to be measured [67]. Establishing the content validity involves evaluation by an expert panel [68]. Construct validity is the degree to which scores of an instrument are consistent with hypotheses [67]. A central concern of construct validity is linking the observed variables with attributes of abstract, the unobserved theoretical variables. It is best evaluated using confirmatory factor analysis (CFA) as it has the clear advantage of indicating whether the data fit the hypothesised factor structure. These hypothesised factor structures were identified from previous studies [28, 30, 31, 34]. Model fit was evaluated using indices of fit, including the χ2 test, comparative fit index (CFI), Tucker Lewis Index (TLI), Root Mean Square Error of Approximation (RMSEA) and Standard Root Mean Square Residual (SRMR). Goodness-of-fit was evaluated by the following criteria: CFI > 0.9, TLI > 0.9, RMSEA < 0.08, SRMR < 0.08 [67][69][47].

Convergent validity looks at the degree to which there is conceptual convergence. The C-EDE-Q was used to compare with the C-DEPS-R, which has good internal consistency, and is regarded as one of the gold standards for measuring eating disorder pathology. Convergent validity was analysed by Spearman’s rho correlation coefficient. A Spearman’s rho correlation coefficient of over 0.7 indicates equivalent psychometric properties of the scales and they are likely measuring similar phenomena [70].

Divergent validity tests whether an instrument measures a different construct other than the one intended [71]. It is demonstrated by having a no or low correlation coefficient between two tests which are known to measure different constructs. In this study, divergent validity was investigated by the correlation between the C-DEPS-R and C-DES-20.

### Statistical analysis

Internal consistency was assessed by the McDonald’s omega in the validation study. Confirmatory factor analysis was performed to examine the constructs of the C-DEPS-R with the use of R Package lavaan) [46]. The weighted least square mean and variance (WLSMV) adjusted estimation method using a polychoric correlation matrix was used in the CFA model. Goodness-of-fit was evaluated by the following criteria: Comparative fit index (CFI) > 0.9; Tucker Lewis Index (TLI) > 0.9, Root Mean Square Error of Approximation (RMSEA) < 0.08 and Standard Root Mean Square Residual (SRMR) < 0.08 [47]. Modification index was considered during the process of finding the optimal result of CFA model by adding the correlation of error terms [48].

The test-retest reliability was determined by a signed-rank test and Spearman’s rho correlation coefficient. Convergent validity between the C-DEPS-R and C-EDE-Q was investigated by Spearman’s correlation. Divergent validity was investigated by comparing the C-DEPS-R with C-DES-20. To investigate the optimal cut-off point of the total score of the C-DERS-R to have eating disorder, a receiver operating characteristic (ROC) curve and the highest value of Youden index [49] of all possible cut-off scores were applied.

Correlation analyses were conducted for investigating the association between the C-DEPS-R and other parameters including e.g. age, BMI, hbA1c, etc. Analyses were performed using IBM SPSS Statistics for Windows, Version 26.0 (IBM Corp., Armonk. NY, USA computer software). For continuous data, median and mean were used to present the sociodemographic and clinical data of the study sample depending on the skewness, and analysed by the Mann-Whitney U test and Kruskal-Wallis H test (for multiple groups) to assess differences in variables. For categorical data, number and percentage was presented and analysed by the Chi-square test or Fisher’s Exact test. Spearman’s Rho correlation coefficient was used for correlation analyses between the C-DEPS-R scores and other clinical factors. Correlation coefficients were interpreted with reference to the recommendations by Mukaka [72] that r above 0.70 (or below − 0.70) indicated a high correlation, r = 0.50 to 0.70 (or -0.50 to -0.70) indicated a moderate correlation, r = 0.30 to 0.50 (or -0.30 to -0.50) indicated a low correlation and r = 0.00 to 0.30 (or 0.00 to -0.30) indicated a negligible correlation.

## Results

### Characteristics of the participants

Of the 228 participants of T1DM, 108 (47.4%) were female and 120 (52.6%) were male. The mean age was 38 (SD = 12.92), 116 (50.9%) participants were single, 105 (46.1%) were married and seven (3.1%) were divorced. The mean age of onset was 22.56 years (SD = 12.14), mean duration of T1DM was 15.7 years (SD = 9.02). The median BMI was 22.13 (IQR 19.96–24.92). All the participants had attained junior secondary education level or above, 104 (45.6%) had tertiary or above education level; 140 (61.4%) were either students or clerical workers; 17 (7.5%) of them did manual work; 12 (5.3%) of them did regular night shift work; 22 (9.6%) did irregular shift work while 37 (16.2%) were unemployed or housewives; 181 (79.4%) of the participants were non-smokers, 25 (11.0%) were smokers and 22 (9.6%) were ex-smokers; 157 (68.9%) of them did not do regular exercise, while 71 (31.1%) did regular exercise. The participants also reported their general meal patterns; 165 (72.4%) of the participants had regular meals, while 63 (27.6%) had irregular meals.

Of the 58 participants of T2DM, 26 (44.8%) were female and 32 (55.2%) were male. The mean age was 40 (SD = 16.18). The mean duration of T2DM was 11.68 years (SD = 9.63). The median BMI was 26.98 (IQR 22.75–30.06). Twenty-six participants (44.8%) were either students or clerical workers; six (10.3%) of them did manual work; three (5.2%) of them did regular night shift work; 12 (20.7%) did irregular shift work while 11 (19%) were unemployed or housewives; 42 (72.4%) were non-smokers; eight (13.8%) were smokers and eight (13.8%) were ex-smokers; 38 (65.5%) of them did not do regular exercise, while 20 (34.5%) did regular exercise; 44 (75.9%) of the participants had regular meals, while 14(24.1%) had irregular meals (Table 1).

**Table 1:** Characteristics of the participants in the study

Participants	T1DM (N = 228)	T2DM (N = 58)
	N (%)/Mean (SD)/Median (IQR)	N (%)/Mean (SD)/Median (IQR)
**Sex**, N (%)		
Female	108 (47.4%)	26 (44.8%)
Male	120 (52.6%)	32 (55.2%)
**Age**, Mean (SD)	38.21 (SD 12.92)	40.88 (SD 16.18)
**Occupation**, N (%)		
Student/office hourly work	140 (61.4%)	26 (44.8%)
Manual/labour-intensive work	17 (7.5%)	6 (10.3%)
Regular night-shift work	12 (5.3%)	3 (5.2%)
Irregular shift work	22 (9.6%)	12 (20.7%)
Unemployed or homemaker	37 (16.2%)	11 (19%)
**BMI**, Median (IQR)	22.13 (19.96–24.92)	26.98 (22.75–30.06)
**Meal**, N (%)		
Regular meal	165 (72.4%)	44 (75.9%)
Irregular meal	63 (27.6%)	14 (24.1%)
**Duration (years)**, Mean (SD)	15.70 (SD 9.02)	11.68 (SD 9.63)
**Exercise**, N (%)		
No regular exercise	157 (68.9%)	38 (65.5%)
Regular exercise	71 (31.1%)	20 (34.5%)
**Smoking**, N (%)		
None	181 (79.4%)	42 (72.4%)
Smoker	25 (11.0%)	8 (13.8%)
Ex-smoker	22 (9.6%)	8 (13.8%)

*Note*: The results are presented as the mean if the data were not skewed and as the median if the data were skewed

SD = standard deviation. IQR = interquartile range.

### Psychometric test results

#### Reliability

The McDonald’s omega of the total C-DEPS-R was 0.825 in the T1DM participants. The values of McDonald’s omega of the sub-scales were 0.767 for the subscale of eating habits, 0.753 for thinness, and 0.619 for high blood glucose. The McDonald’s omega of four subscales of EDE-Q were between 0.753 and 0.905. The test-retest reliability for the total score of the C-DEPS-R was good, with a Spearman’s rho correlation coefficient of 0.991 for the total score (Table 2).

**Table 2:** Test-retest reliability

N = 34	p-value (by Wilcoxon signed-rank test)	Spearman’s rho correlation coefficient (r)
Total score	0.224	0.991
Eating habits	0.901	0.96
Thinness	0.115	0.974
High blood glucose	0.763	0.732

*Note*: The result is said to be satisfactory with p-value > 0.05 by signed-rank test and

Spearman’s rho correlation coefficient > 0.7.

#### Validity

##### Construct validity

**Fig. 3:**
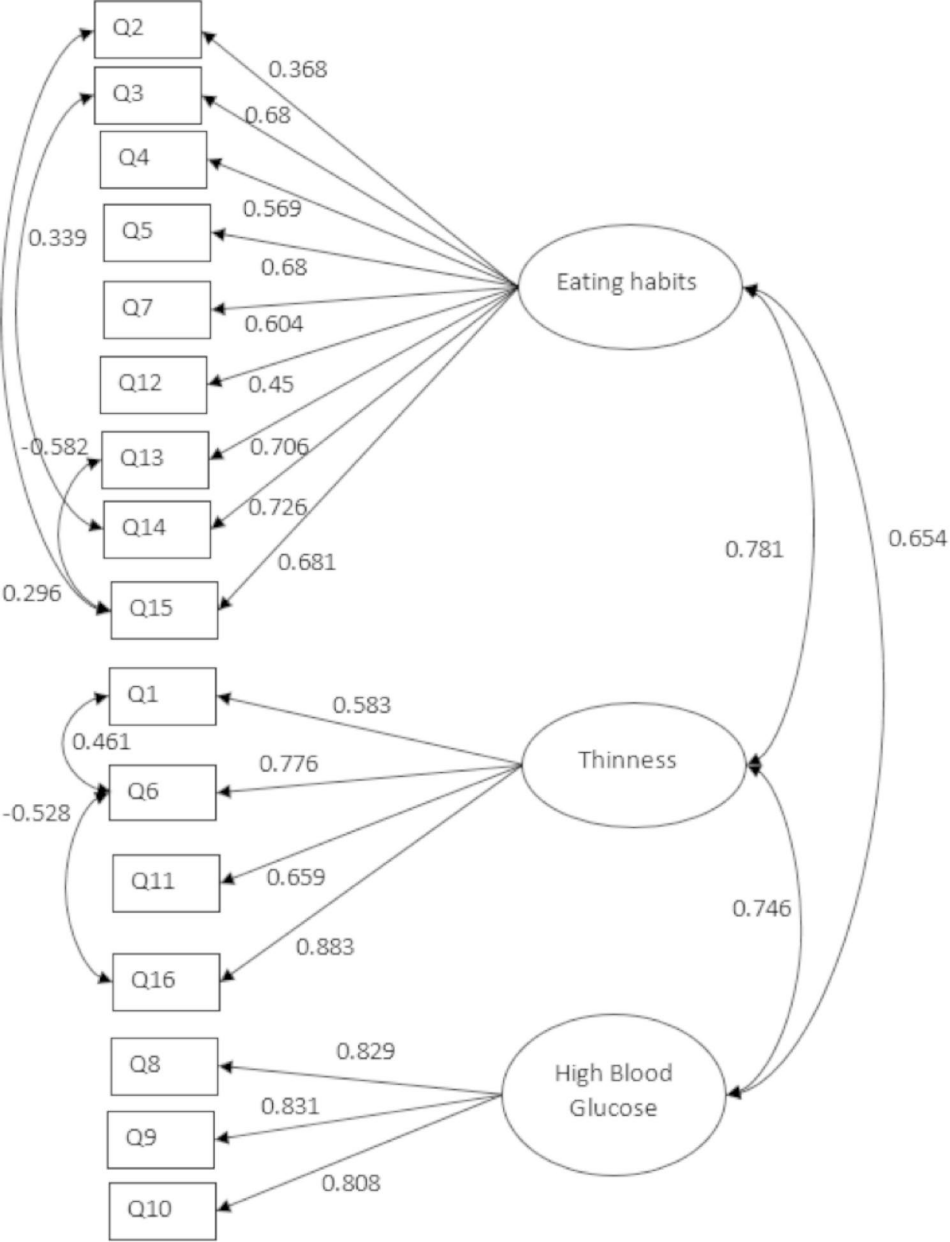
3-factor model of CFA (T1DM participants, N = 228) Note: Rectangles represent observed variables (items in the C-DEPS-R) Ellipses represent latent variables Double-headed arrows are symbols for a correlation The values between the observed and latent variables represent standardised factor loadings The values next to the arrows between the latent variables are standardised regression weights

Model fit was evaluated using indices of fit, including the χ2 test, comparative fit index (CFI), Tucker Lewis Index (TLI), Root Mean Square Error of Approximation (RMSEA) and Standard Root Mean Square Residual (SRMR). A one-factor model, three-factor model, four-factor model, and five-factor model were tested for the confirmatory factor analysis [50]. Due to the similar wordings under “Eating Habits” and “Thinness”, the correlated errors were considered to avoid the under-identification of the model [50]. The three-factor model provided good fit with our data (Fig. 3) (Table 3).

**Table 3:** Confirmatory factor analysis for the three-factor model

Model	χ^2^ (Chi-square)	d.f.	CFI	RMSEA (90% CI)	TLI	SRMR
**Three-factor model **	224.369	101	0.935	0.073 (0.06–0.086)	0.923	0.1
**Three-factor model** **-error term correlated**	165.84	96	0.963	0.041 (0.022–0.057)	0.954	0.088

*Note*: Goodness-of-fit was evaluated by the following criteria: comparative fit index (CFI) > 0.9; Tucker Lewis index (TLI) > 0.9, root mean square error of approximation (RMSEA) < 0.08 and standard root mean square residual (SRMR) < 0.08 [47]

##### Convergent validity

The scores of the C-DEPS-R and the global scores of the C-EDE-Q were investigated with Spearman’s correlations coefficient. There was good convergent validity of the C-DEPS-R with C-EDE-Q with a correlation coefficient of 0.616 (p-value < 0.001).

##### Divergent validity

The scores of the C-DEPS-R and the scores of the C-DES were investigated with Spearman’s correlations coefficient. The correlation coefficient of the C-DEPS-R total scores and C-DES was − 0.235 (p-value < 0.001), which demonstrated divergent validity as the correlation is negligible [72].

### Assessments scoring

The median C-DEPS-R scores were 12 and 12.5 in T1DM and T2DM participants, respectively. The median C-EDE-Q global scores was 0.36 and 0.38 in T1DM and T2DM participants, respectively, and there was no significant difference (Table 4).

**Table 4:** C-DEPS-R scores of the T1DM and T2DM participants

	T1DM participants (N = 228)	T2DM Participants (N = 58)			
	N (%) / Median (IQR)	N (%) / Median (IQR)	p-value		Effect size
**CB-SCID-I/P (H)** SCID (H) -ve	213 (93.4%)	57 (98.3%)		0.208^f^	0.085 (negligible)
SCID (H) + ve	15 (6.6%)	1 (1.7%)			
**C-DEPS-R total score**	12 (6.25-16)	12.5 (5-16.25)		0.627^u^	0.029 (low)
**C-EDE-Q global score**	0.36 (0.12–1.03)	0.38 (0.11–1.13)		0.941^u^	0.004 (very low)

*Note*:

SCID (H) -ve = no diagnosis by CB-SCID-I/P Module H

SCID (H) + ve = eating disorders diagnosed by CB-SCID-I/P Module H

C-DEPS-R = The traditional Chinese Diabetes Eating Problem Survey-Revised

C-EDE-Q = The Chinese version Eating Disorder Examination Questionnaire

Effect size: Cramer’s V (Categorical variables), Rosenthal correlation (Continuous variables with 2 groups comparison), Epsilon square (Continuous variables with multiple groups comparison)

^f^ Fisher’s exact test; ^u^ Mann‒Whitney U test

#### Cut-off score of the C-DEPS-R

By applying a receiver operating characteristic (ROC) curve, the optimal cut-off point of the total score of the C-DEPS-R to having an eating disorder was determined by the highest value of Youden index [49]. A new cut-off score of 24 was found through comparison with active diagnosis of eating disorders by CB-SCID-I/P module H, and it demonstrated higher specificity, positive predictive value and accuracy when compared with the predetermined cut-off score of 20 (Supplementary Tables 1 and Supplementary Fig. 1).

### Prevalence of DEB and ED found in the study

Of the T1DM participants in the KEC, 13.2% (95% CI 8.8%-17.5%) scored above the cut-off score for the C-DEPS-R, 7.5% (95% CI 4-10.9%) scored above the cut-off score for the C-EDE-Q, and 4.4% (95% CI 2.1%-7.9%) were assessed as having eating disorders at the moment of recruitment by module H of CB-SCID-I/P with breakdown diagnoses (Table 5).

**Table 5:** Psychometric test results in the different sample groups

	T1DM (N = 228) N (%)	T2DM (N = 58) N (%)	All (N = 286) N (%)
C-DEPS-R score			
<24	198 (86.8%)	53 (91.4%)	251 (87.8%)
≥24	30 (13.2%)	5 (8.6%)	35 (12.2%)
**C-EDE-Q Global score or any subscale**			
<4	211 (92.5%)	55 (94.8%)	266 (93%)
≥4	17 (7.5%)	3 (5.2%)	20 (7%)
**CB-SCID-I/P (H)** SCID (H) -ve	213 (93.4%)	57 (98.3%)	270 (94.4%)
SCID (H) + ve Non-active/in full remission	5 (2.2%)	1 (1.7%)	6 (2.1%)
Diagnosis from SCID (H)	4 out of 5 (80%) with BN (non-purging type) 1 out of 5 (20%) with AN (binge/purge type)	BED	
SCID (H) + ve Active, or in partial remission	10 (4.4%)	0 (0%)	10 (3.5%)
Diagnosis from SCID (H)	6 out of 10 (60%) with BN (purging type) 4 out of 10 (40%) with BN (non-purging type)		

*Note*:

C-DEPS-R = The traditional Chinese Diabetes Eating Problem Survey-Revised

C-EDE-Q = The Chinese version Eating Disorder Examination Questionnaire

SCID (H) -ve = no diagnosis by CB-SCID-I/P Module H

SCID (H) + ve = eating disorders diagnosed by CB-SCID-I/P Module H

### Sex effect and age effect

In the T1DM group, females scored higher on the C-DEPS-R (p = 0.001). Males with T2DM scored higher on the C-DEPS-R (p = 0.031) (Supplementary file Table 2). There was no significant difference between the median score (p = 0.716) on the C-DEPS-R and the percentage above the cut-off score (p = 0.811) in the T1DM youth and adult groups (Supplementary file Table 3).

### Correlation and comparison of the C-DEPS-R score and other clinical variables

There were significantly higher C-DEPS-R scores in participants diagnosed with eating disorders by CB-SCID-I/P (Table 6).

**Table 6:** Comparison of the C-DEPS-R score and diagnosis from CB-SCID-I/P Module H

	N	T1DM (N = 228)				
		C-DEPS-R score				
		Median (IQR) / correlation coefficient	p value		Effect size	
**CB-SCID-I/P (H)**			^***^<0.001^ h^		0.089 (Moderate)	
SCID(H) -ve	213	11 (6–16)				
SCID (H) + ve but non-active/ in full remission	5	14 (12.5–17)				
SCID (H) + ve, active or in partial remission	10	29 (22.25-32)				

*Note*: ^***^p$<0.001,$Data were analysed by ^h^ the Kruskal‒Wallis H test

SCID (H) -ve = no diagnosis by CB-SCID-I/P Module H

SCID (H) + ve = eating disorders diagnosed by CB-SCID-I/P Module H

IQR = Interquartile Range

Effect size: Cramer’s V (Categorical variables), Rosenthal correlation (Continuous variables with 2 groups comparison), Epsilon square (Continuous variables with multiple groups comparison)

Table 7 shows a positive correlation between the C-DEPS-R score and BMI (r = 0.318, p < 0.001), weak negative correlation with age of DM onset (r= -0.181, p = 0.006). The C-DEPS-R score was weakly correlated with the triglyceride level (r = 0.128, p < 0.01), and with the total cholesterol level (r = 0.149, p < 0.05). T1DM participants who scored higher on the C-DEPS-R had more occurrence of DKA. There were higher scores on the C-DEPS-R in patients with continuous glucose monitoring.

**Table 7:** Correlation and comparison between C-DEPS-R scores and other clinical variables in T1DM (N = 228)

	N (%)/ Mean (SD)/ Median (IQR)	T1DM C-DEPS-R Median score (IQR) / correlation coefficient(r)		p value	Effect size	
Occupation				^*^0.043^ h^	0.043 (Moderate	
Student/office hourly work	140 (61.4%)		13 (8–17)			
Manual/labour-intensive work	17 (7.5%)		10 (3.5–14)			
Regular night-shift work	12 (5.3%)		13 (4.25–15.5)			
Irregular shift work	22 (9.6%)		13 (8.75–17.75)			
Unemployed or homemaker	37 (16.2%)		8 (4–13)			
**BMI**	22.13 (19.96–24.92)		r = 0.352	^***^<0.001^r^	--	
**Meal**				^*^0.016^u^	0.160 (low)	
Regular meals	165 (72.4%)		11 (6-15.5)			
Irregular meals	63 (27.6%)		14 (9–18)			
**Age of DM onset** (years)	22.56 (SD 12.14)		r= -0.181	^**^0.006^r^	^−−^	
**Duration** (years)	15.70 (SD 9.02)		r = 0.094	0.158^r^	--	
**Exercise**				0.843^u^	0.013 (very low)	
No regular exercise	157 (68.9%)		12 (6.5–16)			
Regular exercise	71 (31.1%)		12 (6–17)			
**Smoking**				0.285^ h^	0.011 (weak)	
None	181 (79.4%)		12 (7–17)			
Smoker	25 (11.0%)		11 (5–15)			
Ex-smoker	22 (9.6%)		11 (3-14.5)			
Laboratory parameters HbA1c	7.75 (6.9–8.7)	r = 0.118			0.076^r^	--
Urea	5.05 (4.2–6.08)	r= -0.032			0.626^r^	--
Creatinine	70 (59.25-79)	r= -0.098			0.139^r^	--
ALP	70.5 (58–87)	r= -0.044			0.506^r^	--
ALT	17 (12–25)	r= -0.03			0.655^r^	--
FG	8.4 (6.13–10.9)	r = 0.047			0.483^r^	--
TG	0.8 (0.6-1)	r = 0.128			^**^0.006^r^	--
Chol	4.5 (3.9–5.1)	r = 0.149			^*^0.024^r^	--
HDL	1.6 (1.3–1.9)	r= -0.009			0.888^r^	--
LDL	2.5 (2-2.8)	r = 0.097			0.149^r^	--
**DKA history**					^*^0.03^ h^	0.031 (weak)
Nil	111 (48.7%)	11 (5–17)				
DKA at the onset	56 (24.6%)	10 (6-15.75)				
Impending DKA/DKA/Repeated DKA	61 (26.8%)	14 (9.5–17.5)				
**Complications**					0.091^u^	0.112 (very low)
No	77 (33.8%)	10 (5–16)				
Yes	151 (66.2%)	13 (7–17)				
**SCID (H)** SCID (H) -ve	213 (93.4%)	11 (6–16)			^***^<0.001^u^	0.278 (low)
SCID (H) + ve	15 (6.6%)	24 (14–32)				
**Peer support program**					0.317^u^	0.066 (very low)
Not joined	141 (61.8%)	11 (6-16.5)				
Joined	87 (38.2%)	13 (8–16)				
Continuous glucose monitoring (CGM)			^**^0.003^u^	0.200 (low)		
No	95 (41.7%)	10 (5–15)				
Yes	133 (58.3%)	13 (9–18)				
**Insulin type** ^**+**^			0.724^ h^	0.009 (very low)		
Pump	7 (3.1%)	14 (9–18)				
Short/rapid-acting alone ^**+**^	1 (0.4%)	32				
Intermediate/long-acting alone	7 (3.1%)	11 (7–15)				
Mixed short/rapid with inter- mediate/long-acting	124 (54.4%)	11 (6–16)				
Intermediate/long-acting with oral hypoglycaemic drugs	6 (2.6%)	16 (9.75–26.5)				
Mix (intermediate or long-acting along) with oral drugs	83 (36.4%)	12 (6–17)				

*Note*: ^**^p<0.01. Data were analysed by ^u^ the Mann‒Whitney U test / ^h^ the Kruskal‒Wallis H test/ ^r^ Spearman’s rho correlation. Results were presented in mean if data not skewed, and presented in median if data was skewed

SD = Stand deviation. IQR = Interquartile Range.

Note: ^+^Insulin type “Short/rapid acting alone” was excluded in the analysis part due to small sample size.

Effect size: Cramer’s V (Categorical variables), Rosenthal correlation (Continuous variables with 2 groups comparison), Epsilon square (Continuous variables with multiple groups comparison).

There was no significant correlation between the C-DEPS-R total scores and HbA1c, but when the correlation was explored with the C-DEPS-R subscales, eating habits and high blood glucose subscales exhibited weak correlations with HbA1c (r = 0.166, p < 0.05 and r = 0.132, p < 0.05), respectively.

### Comparison of T1DM and T2DM participants

T1DM participants had a higher occurrence of DKA but no significant difference in CB-SCID-I/P Module H results or the C-DEPS-R or C-EDE-Q scores (Table 8).

**Table 8:** Comparison between T1DM participants and T2DM participants

	T1DM (N = 228)	T2DM (N = 58)					
	N (%) / Median (IQR)	N (%) / Median (IQR)		p value		Effect size	
BMI	22.13 (19.96–24.92)	26.98 (22.75–30.06)		^***^<0.001^u^		0.340 (low)	
**Laboratory parameters**							
HbA1c	7.75 (6.9–8.7)	7.90 (6.83-9.00)		0.687^u^		0.024 (very low)	
Ur	5.05 (4.2–6.08)	5.25 (4.48–7.50)		0.086^u^		0.102 (very low)	
Cr	70 (59.25-79)	76.00 (59.75–90.75)		0.058^u^		0.112 (very low)	
ALP	70.5 (58–87)	71.50 (56.75-89.00)		0.978^u^		0.002(very low)	
ALT	17 (12–25)	26.00 (15.00-39.25)		^***^<0.001^u^		0.216 (low)	
FG	8.4 (6.13–10.9)	7.55 (6.48–10.95)		0.999^u^		< 0.001(very low)	
TG	0.8 (0.6-1)	1.30 (0.78-2.00)		^***^<0.001^u^		0.308 (low)	
Chol	4.5 (3.9–5.1)	4.40 (3.70-5.00)		0.302^u^		0.061(very low)	
HDL	1.6 (1.3–1.9)	1.20 (1.00-1.43)		^***^<0.001^u^		0.341 (low)	
LDL	2.5 (2-2.8)	2.40 (1.90–2.95)		0.526^u^		0.038(very low)	
**DKA status**				^**^0.005^f^		0.227 (Large)	
Nil	111 (48.7%)		44 (75.9%)				
DKA at onset	56 (24.6%)		5 (8.6%)				
Impending DKA	10 (4.4%)		2 (3.4%)				
DKA	47 (20.6%)		7 (12.1%)				
Repeated DKA	4 (1.8%)		0 (0%)				
**Complications**				0.055^c^		0.114 (Small)	
No	77 (33.8%)		12 (20.7%)				
Yes	151 (66.2%)		46 (79.3%)				
**CB-SCID-I/P (H)**				0.208^f^		0.085 (Negligible)	
SCID (H) -ve	213 (93.4%)		57 (98.3%)				
SCID (H) + ve	15 (6.6%)		1 (1.7%)				

*Note*: ^***^p$<0.001,$^**^p$<0.01$, ^*^p$<$0.05. Continuous variables are analysed by the ^t^ independent-samples t test or ^u^ Mann‒Whitney U test. Categorical variables were analysed by the ^c^ Pearson chi-square test or ^f^ Fisher’s exact test. Effect size: Cramer’s V (Categorical variables), Rosenthal correlation (Continuous variables with 2 groups comparison), Epsilon square (Continuous variables with multiple groups comparison)

## Discussion

### Psychometric properties of the traditional C-DEPS-R

The traditional C-DEPS-R exhibited good internal consistency with McDonald’s omega of 0.825 in the T1DM participants. The subscales of the C-DEPS-R demonstrated acceptable internal consistency, with McDonald’s omega of 0.767 for the subscale of eating habits, 0.753 for thinness, and 0.619 for high blood glucose. A lower internal consistency for the high blood glucose subscale was seen in other validation studies as well. The Cronbach’s alpha of the high blood glucose subscales was 0.596 in the Italian study (Pinna et al., 2017), and 0.48 in the Norwegian study [28]. The lower internal consistency could be a result from lower number of questions, suboptimal correlation of inter-relatedness between items. More related items assessing the ideas of maintaining higher blood glucose could be added in order to improve the internal consistency of the subscale [73]. The test-retest reliability for the traditional C-DEPS-R was good. The CFA revealed good fits in the three-factor model. The three factors relating to the dimensions of “maladaptive eating habits” (factor 1), “preoccupation with thinness” (factor 2) and “maintaining high glucose” (factor 3) were confirmed. This is in line with the Norwegian study, the Italian study and the Mandarin Chinese study [28][31][34]. The significant positive correlation of 0.616 (p < 0.001) between C-DEPS-R scores and C-EDE-Q demonstrated convergent validity. The result is comparable to that of the Norwegian study [28] which yielded a positive correlation of the Norwegian DEPS-R with EDE-Q of 0.68 in females (p = 0.001) and 0.52 in males (p = 0.01), while the correlation of the German DEPS-R and EDE-Q [29] was 0.70 overall (p < 0.001). The negligible correlations (r= -0.235, p < 0.001) between C-DEPS-R scores and C-DES-20 scores demonstrated divergent validity.

Overall, the traditional Chinese-DEPS-R demonstrated a good degree of reliability and good psychometric properties for use in the local population. A new cut-off score of 24 for the traditional C-DEPS-R was determined in this study. It demonstrated higher sensitivity and specificity than the predetermined cut-off score of 20 applied by local traditional Chinese users (Supplementary Table 1).

The traditional C-DEPS-R can be used as an assessment tool in DM clinics for identifying individuals with higher risk of DEB in T1DM. As mentioned previously about the difference on psychopathology of generic ED and DEB in T1DM, SCID assessment could not cover DM-specific DEB symptoms. T1DM with DEB who does not meet the required frequency and severity of diagnosis of ED in SCID will be missed. It also requires specific training to the administrator of SCID on assessing insulin manipulation as compensatory behaviour and assessing the subjective overeating during hypoglycaemic episodes. The traditional C-DEPS-R is easily self-administered, can be completed within a short period of time of around 10 min, it measures DEB in T1DM patients that traditional assessments, e.g. SCID and EDE-Q may not be the most suitable tool for use. The traditional C-DEPS-R was also developed in an electronic version which requires less manpower and resources. It also supports automation in data input and handling, helps promote paperless medical care and facilitates patient care even in situations like the COVID-19 pandemic.

### Prevalence of DEBs found in T1DM patients in the KEC

Of the T1DM participants, 13.2% (95% CI 8.8%-17.5%) scored above the cut-off score of 24 for the traditional C-DEPS-R, 7.5% (95% CI 4-10.9%) scored above the cut-off score for the C-EDE-Q, and 4.4% (95% CI 2.1%-7.9%) were assessed as exhibiting EDs (all had a diagnosis of BN) by module H of the CB-SCID-I/P at the moment of recruitment. The selection filter of this study, i.e., the older age group, the exclusion of patients with concurrent medical illness, etc., may cause an underestimation of the true prevalence. The prevalence of ED diagnosed in T1DM (4.4%) was higher than that of the general population (1.69%) [51]. The results found in this study are comparable to findings in the meta-analysis by Young et al. [13], with a 2.8-7% ED prevalence in T1DM patients.

### Sex difference and other correlations found in the study

T1DM females demonstrated higher scores in the C-DEPS-R, and this finding was comparable to those in other studies [28, 31, 32]. However, T2DM males scored higher on the C-DEPS-R than females in this study, which is incompatible with the results found in a multicentre study [52]. The possible reasons may include the small sample size of T2DM participants in this study and the possible sampling bias of recruiting T2DM participants from specialised DM clinics.

The study found a significant correlation between the presence of DEB and DKA. People with higher BMI are considered at risk of developing DEBs [53], and a higher BMI is associated with higher scores for thinness, body dissatisfaction, and lower self-esteem [54]. This study also found a correlation between DEB and the use of CGM. Hanlan et al. [55] suggested the possibility that patients may think that they can keep their blood glucose elevated safely with the presence of CGM.

There was no significant correlation found between the C-DEPS-R total scores and HbA1c in this study. Lv et al. [34] found a significant correlation of HbA1C with the C-DEPS-R score in the youth group (r = 0.459, p < 0.001), but the correlation was insignificant in the adult group (r = 0.215, p = 0.097).

There are several proposed reasons for the difference found. First, a limited number of young participants were recruited for this study. Research has found that HbA1c increases with age [56], and the diagnostic efficiency of HbA1c also decreases with age [57]. Second, Asians were found to have higher HbA1c levels than Western populations [58, 59], which reflects that ethnicity could be a factor affecting HbA1c levels. Third, some other factors could potentially affect the HbA1c level and glycaemic control of patients, especially depressive symptoms [60] and stress and anxiety symptoms [61].

The study was carried out during the COVID-19 pandemic, there were studies reflected the increases in snacking and emotional eating in the pandemic [74]. There are difficulties in maintaining weight management behaviours due to different psychosocial reasons [75]. The closing down of hospital clinics and reduction in DM supporting activities in the health care system during the COVID-19 social distancing policy could have impact on DM patients’ glycaemic control. The COVID-19 situation could potentially affect the findings in this study regarding the severity of DM-related complications and the prevalence of eating problems.

### Management of ED and DEB in T1DM

To manage T1DM with ED, the NICE guideline suggests collaboration of the eating disorder team and the diabetes team and to set a lower threshold for blood glucose and ketone monitoring, educate patients and caregivers and address insulin misuse in psychological treatments. This suggests a gradual increase in dietary carbohydrates, gradual resumption of the insulin dosage in patients and close monitoring of blood potassium levels. DM patients with BN should be aware of glucose toxicity, insulin resistance, ketoacidosis and oedema [62, 63]. A systematic review found mixed results for the effectiveness of psychological treatments for ED in T1DM populations [64].

The Joslin Diabetes Center has recommended early routine screening for DEB in T1DM patients to promote familial co-management of diabetes control and to reduce the diabetes-related conflicts in the family [4]. Moreover, it recommends the promotion of self-esteem and body acceptance, together with access to a multidisciplinary team, including endocrinologists, nurse educators, nutritionists, and mental health providers. It also recommends avoiding too-intense glycaemic controls as the early treatment goal and states that therapists should set small incremental goals that patients can feel capable of working towards [4, 65].

### Limitations

Patients who were followed up in family medicine clinics or in the private sector were not recruited. The study only involved one cluster area in Hong Kong. It also did not include patients younger than 15 years, although EDs can develop in early adolescence. The SCID-5 or DISC-5 was not available in Chinese, while there was no adaptation for the CB-SCID-I/P made in this study for DSM-5 criteria. Moreover, patients with other types of DM were not investigated. Not enough age-matched T2DM were included in the study as there are not many young T2DM patients on insulin. Most T2DM patients with relatively stable glycaemic control are followed up in family medicine clinics; the selection of T2DM samples in specialised DM clinic in this study may affect the observation of T2DM and DEB relations.

### Future research directions

The validation studies can be extended to younger groups of patients, T2DM patients and other types of DM patients. The disease model of DEBs in T1DM and the related risk factors should be explored in future studies.

## Conclusion

The traditional C-DEPS-R is a self-rated, time-efficient, reliable and valid tool for the screening of DEBs in T1DM patients in the Hong Kong Chinese population. It can be a useful tool for DM patient care and research. The higher prevalence of EDs in T1DM patients than in the general population reflects a service need in this group of patients.

## Electronic supplementary material

Below is the link to the electronic supplementary material.

Supplementary Material 1 Table 1

Supplementary Material 2 Figure 1

Supplementary Material 3 Table 2

Supplementary Material 4 Table 3

## Data Availability

The datasets used and/or analysed during the current study are available from the corresponding author on reasonable request.

## Abbreviations

ALP: Alkaline phosphatase
ALT: Alanine aminotransferase
AN: Anorexia nervosa
AUC: Area Under the Curve
BED: Binge Eating Disorder
BMI: Body Mass Index
BN: Bulimia nervosa
C-DEPS-R: Chinese Diabetes Eating Problem Survey-Revised
C-DES-20: The Chinese Diabetes Empowerment Scale-20
C-EDE-Q: Chinese Eating Disorder Examination-Questionnaire
CB-SCID-I/P: Chinese Bilingual version of the Structured Clinical Interview for DSM-IV-TR Axis I Disorders, Research Version, Patient Edition
CBT: Cognitive behavioural therapy
CFA: Confirmatory factor analysis
CFI: Comparative Fit Index
CGM: Continuous glucose monitoring
Chol: Cholesterol
CI: Confidence interval
d.f.: Degrees of Freedom
DEB: Disordered eating behaviours
DEPS-R: Diabetes Eating Problem Survey-Revised
DES: The Diabetes Empowerment Scale
DKA: Diabetic ketoacidosis
DM: Diabetes Mellitus
DSM: Diagnostic and Statistical Manual of Mental Disorders
DSM-5: Diagnostic and Statistical Manual of Mental Disorders, Fifth Edition
DSM-III: Diagnostic and Statistical Manual of Mental Disorders, Third Edition
DSM-IV: Diagnostic and Statistical Manual of Mental Disorders, Fourth Edition
ED: Eating disorders
EDE: Eating Disorder Examination
EDE-Q: Eating Disorder Examination-Questionnaire
EDNOS: Eating Disorder Not Otherwise, Specified
FG: Fasting glucose
HbA1c: Haemoglobin A1c
HDL: High-density lipoprotein
ICC: Intraclass correlation coefficient
IQR: Interquartile Range
KEC: Kowloon East Cluster
LDL: Low-density lipoprotein
MANTRA: The Maudsley Model of Anorexia Treatment in Adults
MARISPAN: Management of Really Sick Patients with Anorexia Nervosa
NICE: National institute for Health and Care Excellence
QR code: Quick Response code
RMSEA: Root Mean Square Error of Approximation
ROC: Receiver operating characteristic Curve
SCID: Structured Clinical Interview for DSM-IV-TR
SCID-5: The Structured Clinical Interview for DSM-5
SCID-I/P: Structured Clinical Interview for DSM-IV-TR Axis I Disorder, Research Version, Patient Edition
SPSS: Statistical Package for the Social Science
SRMR: Standard Root Mean Square Residual
T1DM: Type 1 diabetes mellitus
T2DM: Type 2 diabetes mellitus
TG: Triglycerides
TKOH: Tseung Kwan O Hospital
TLI: Tucker Lewis Index
UCH: United Christian Hospital
Vr.: Version
WHO: World Health Organization
χ2: Chi-square
