# Supplementary material for: Validation of the traditional Chinese version of the diabetes eating problem survey-revised and study of the prevalence of disordered eating patterns in Chinese patients with type 1 DM
Source: BMC Psychiatry. 2023 May 31;23:382. doi: 10.1186/s12888-023-04744-6 (PMC10230489; doi:10.1186/s12888-023-04744-6)
Supplement: Supplementary file 1 — Supplementary Material 1 Table 1 [file 12888_2023_4744_MOESM1_ESM.docx]

**Supplementary Table 1**

*Cut-off scores*

|  | Cut-off score at 24  (new cut-off) | Cut-off score at 20  (predetermined cut-off) |
| --- | --- | --- |
| **Sensitivity** | 80% (95% CI, 44.4%-97.48%) | 80% (95% CI, 44.4%-97.5%) |
| **Specificity** | 89.9% (95% CI, 85.1%-93.6%) | 87.2% (95% CI, 82%-91.3%) |
| **Positive predictive value** | 26.7% (95% CI, 12.3%-45.9%) | 22.2% (95% CI, 10.1%-39.2%) |
| **Negative predictive value** | 99% (95% CI, 96.4%-99.9%) | 99% (95% CI, 96.3%-99.9%) |
| **Accuracy** | 89.5% (95% CI, 84.7%-93.1%) | 86.8% (95% CI, 81.8%-90.9%) |
| **Area under the curve (AUC)** | 90.6% (95% CI, 79.7%-100%) | 90.6% (95% CI, 79.7%-100%) |

*Note:* CI= Confidence interval
