# Supplementary material for: Validation of the traditional Chinese version of the diabetes eating problem survey-revised and study of the prevalence of disordered eating patterns in Chinese patients with type 1 DM
Source: BMC Psychiatry. 2023 May 31;23:382. doi: 10.1186/s12888-023-04744-6 (PMC10230489; doi:10.1186/s12888-023-04744-6)
Supplement: Supplementary file 2 — Supplementary Material 2 Figure 1 [file 12888_2023_4744_MOESM2_ESM.docx]

**Supplementary Figure 1**

*Receiver operating characteristic curve*

C-DEPS-R score at 24
