# Supplementary material for: Validation of the traditional Chinese version of the diabetes eating problem survey-revised and study of the prevalence of disordered eating patterns in Chinese patients with type 1 DM
Source: BMC Psychiatry. 2023 May 31;23:382. doi: 10.1186/s12888-023-04744-6 (PMC10230489; doi:10.1186/s12888-023-04744-6)
Supplement: Supplementary file 3 — Supplementary Material 3 Table 2 [file 12888_2023_4744_MOESM3_ESM.docx]

**Supplementary Table 2**

*C-DEPS-R scores by sex*

|  | **N** | **T1DM patients (N=228)**  **C-DEPS-R Median score (IQR)** | **p-value** | | **Effect size** | |
| --- | --- | --- | --- | --- | --- | --- |
| **Sex** |  |  | ^***^0.001^u^ | | 0.230 (low) | |
| Female | 108 | 14.00 (9.00-19.00) | |  | |  |
| Male | 120 | 10.50 (5.00-15.00) | |  | |  |

|  | **N** | **T2DM patients (N=58)** | |  | |  |
| --- | --- | --- | --- | --- | --- | --- |
|  |  | **C-DEPS-R Median score (IQR)** | **p-value** | | **Effect size** | |
| **Sex** |  |  | ^*^0.054^u^ | | 0.253 (low) | |
| Female | 26 | 9.00 (4.75-14.00) | |  | |  |
| Male | 32 | 14.00 (6.50-18.50) | |  | |  |

*Note:* ^**^ p$<0.01,$^*^p$<$0.05, ^u^Mann‒Whitney U test
