# Supplementary material for: Validation of the traditional Chinese version of the diabetes eating problem survey-revised and study of the prevalence of disordered eating patterns in Chinese patients with type 1 DM
Source: BMC Psychiatry. 2023 May 31;23:382. doi: 10.1186/s12888-023-04744-6 (PMC10230489; doi:10.1186/s12888-023-04744-6)
Supplement: Supplementary file 4 — Supplementary Material 4 Table 3 [file 12888_2023_4744_MOESM4_ESM.docx]

**Supplementary Table 3**

*Differences in C-DEPS-R scores for the youth and adult groups in T1DM*

|  | **N (N=228)** | **T1DM C-DEPS-R**  **Median score (IQR)** | | | **p value** | | | **Effect size** |
| --- | --- | --- | --- | --- | --- | --- | --- | --- |
| **Age group** |  |  | | | 0.716^u^ | | | 0.024 (very low) |
| Youth group (15-24 y.o) | 42 | 11 (7.75-16.25) |  | | |  | | |
| Adult group (25-64 y.o) | 186 | 12 (6-16.25) | |  | | |  | |

*Note:* ^**^ p$<0.01,$^*^p$<$0.05, ^u^ Data was analysed by Mann-Whitney U test. y.o = year-old.

IQR= Interquartile Range

Effect size: Cramer’s V (Categorical variables), Rosenthal correlation (Continuous variables with 2 groups comparison), Epsilon square (Continuous variables with multiple groups comparison).
